# Performance in a variable world: using Jensen’s inequality to scale up from individuals to populations

**DOI:** 10.1093/conphys/coz053

**Published:** 2019-09-04

**Authors:** Mark Denny

**Affiliations:** Hopkins Marine Station of Stanford University, 120 Ocean View Blvd., Pacific Grove, California, 93950 USA

**Keywords:** Jensen's inequality, scaling, temperature variation, thermal performance curves, 1/*f* noise

## Abstract

Body temperature affects plants’ and animals’ performance, but these effects are complicated by thermal variation through time within an individual and variation through space among individuals in a population. This review and synthesis describes how the effects of thermal variation—in both time and space—can be estimated by applying a simple, nonlinear averaging scheme. The method is first applied to the temporal variation experienced by an individual, providing an estimate of the individual’s average performance. The method is then applied to the scale-dependent thermal variation among individuals, which is modelled as a 1/*f*-noise phenomenon. For an individual, thermal variation reduces average performance, lowers the temperature of maximum performance (*T_opt_*) and contracts the range of viable temperatures. Thermal variation among individuals similarly reduces performance and lowers *T_opt_*, but increases the viable range of average temperatures. These results must be viewed with caution, however, because they do not take into account the time-dependent interaction between body temperature and physiological plasticity. Quantifying these interactions is perhaps the largest challenge for ecological and conservation physiologists as they attempt to predict the effects of climate change.

## Teaser

Body temperature affects plants’ and animals’ performance, but temperature varies through time within an individual and through space among individuals. Theory developed here predicts that, for an individual, variation reduces performance and contracts the range of viable temperatures. By contrast, variation among individuals reduces performance but increases the viable range.

## Introduction

Temperature affects all aspects of life. The speed of chemical reactions increases with increasing temperature, affecting the rates of photosynthesis, metabolism, growth and locomotion (Somero et al., 2017). The mechanical properties of biological structural materials change with changes in temperature (Gosline, 2018), as do the rates of evaporation and condensation and the solubility of important gases (Denny, 1993). The ability to obtain food, escape predation or compete for critical resources often varies with temperature, and, if this variation differs among species, changes in temperature can thereby affect community composition (for a synthesis, see Harley, 2013). Compounding our interest in these effects, globally averaged temperature is rising due to the increasing concentration of carbon dioxide and other greenhouse gases in the atmosphere (IPCC, 2013).

The current emphasis on latitudinal- and global-scale changes in average temperature can, however, overshadow the importance of temperature measured at the scale of individual organisms. Populations may move in response to latitudinal shifts in average temperature, and species may evolve in response to thermally influenced shifts in selection pressure, but these changes manifest because temperature affects the individuals that form those populations and species. In this review and synthesis, I explore how temporal variation in temperature affects the performance of individual organisms, and how spatial variation in temperature among individuals determines average population performance; both are issues of importance to conservation biology.

Most organisms experience some change in temperature during their lifetime. In terrestrial environments, direct exposure to sunlight sets the stage for rapid warming during the day, and the infrared transparency of air allows organisms to cool rapidly at night (Gates, 1980; Denny, 2016). Consequently, many terrestrial organisms experience daily changes in body temperature of 20–30°C and even greater fluctuation across seasons. Furthermore, the low thermal conductivity and heat capacity of air and the possibility of evaporative cooling mean that for many terrestrial organisms body temperature (what ecophysiologists refer to as operational temperature) can differ substantially from air temperature (Gates, 1980). For instance, the flight muscles of bees (Heinrichs, 1979) and the reproductive structures of the voodoo lily (Meeuse, 1966) can be 35–45°C above ambient air temperature, and evaporative cooling can chill seaweeds and desert herbs 5–8°C below air temperature (Bell 1995, Potter et al., 2009).

Because water has an unusually large heat capacity and a high thermal conductivity, temperature fluctuations in aquatic environments tend to be less dramatic in both space and time than those in terrestrial environments. With the exception of a few animals (such as tunas, marine mammals), the body temperature of pelagic organisms is the same as that of the water in which they are immersed. Consequently, deep-sea organisms are likely to experience changes of less than 1°C in their lifetimes. However, even in the thermally conservative aquatic environment, some organisms encounter substantial variation in temperature. For example, a wide variety of ectothermic marine animals migrate hundreds of vertical meters daily, moving from the cold water below the thermocline (where they spend the day), up to the warm surface waters at night, often encountering a swing of 10°C or more along the way (Denny, 2008). When exposed to bright sunlight, shallow-water corals can be heated to 1.5°C above ambient water temperature (Fabricius, 2006; Jimenez et al., 2008), and the arrival of internal waves can impose fluctuations of 8–10°C in the course of a few hours (Leichter et al., 2006). In rivers downstream from dams, the episodic release of water from a reservoir can abruptly lower the temperature by 8–12°C (Olden and Naiman, 2010).

**Figure 1:**
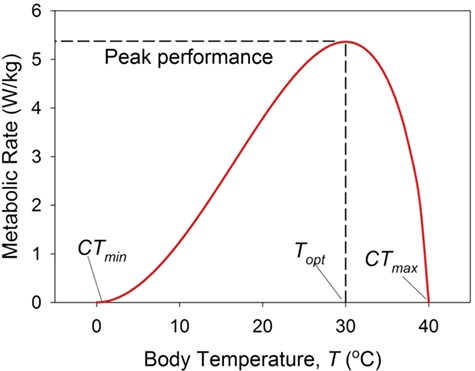
A representative nominal thermal performance function: *P*(*T*), metabolic rate as a function of body temperature *T*. The organism’s thermal breadth is bracketed by its minimum viable temperature (*CT_min_*) and it maximum viable temperature (*CT_max_*). Peak performance occurs at *T_opt_*. Note that the curve is concave downward except for temperatures neat *CT_min_*.

Given that temperature governs virtually all aspects of life, how do these thermal fluctuations affect the performance of organisms and populations?

### The thermal performance function

The pattern in which an organism’s performance varies as a function of body temperature is traditionally documented as a thermal performance curve or, equivalently, a thermal performance function (Fig. 1; Huey and Stevenson, 1979). Performance in this context can take on many guises: e.g. heart rate, metabolic rate, speed of locomotion, growth rate or reproductive output. There is a minimum critical temperature (*CT_min_*) below which performance is unacceptably low. For temperatures above *CT_min_*, performance increases, reaching a maximum at *T_opt_*, and then decreases to a maximum critical temperature (*CT_max_*) above which performance again sinks below the acceptable level. For biochemical and physiological rate data (e.g. metabolic rate), the performance curve is skewed to the left: performance gradually accelerates as temperature increases above *CT_min_* (according to the Boltzmann–Arrhenius relationship), decelerates to a maximum value at *T_opt_* and then rapidly declines to *CT_max_* (Angilletta, 2009). For more integrative processes (e.g. reproductive rate), the curve is more symmetrical about *T_opt_* (Knies and Kingsolver, 2010). The range of temperatures over which an organism’s performance is viable—the thermal breadth (*CT_max_* – *CT_min_*)—varies among species. Organisms with a large thermal breadth are thermal generalists (eurytherms); those with narrow breadth are thermal specialists (stenotherms).

Thermal performance functions have been measured for a wide variety of species for a broad range of performance metrics (Angiletta, 2009). In this review, I use metabolic rate as a heuristic example, a choice that is convenient for two reasons. First, metabolic rate is a key factor in many other aspects of performance: growth rate, reproductive rate and speed of locomotion, for instance, are all likely to increase as metabolic rate increases. Second, unlike some other metrics of performance, metabolic rate provides easily defined criteria for *CT_min_* and *CT_max_*. With few exceptions, an organism can survive only if its metabolic rate is greater than zero. Thus, for values less than *CT_min_* or greater than *CT_max_*, I assume that the organism dies and performance is unambiguously zero.

**Figure 2:**
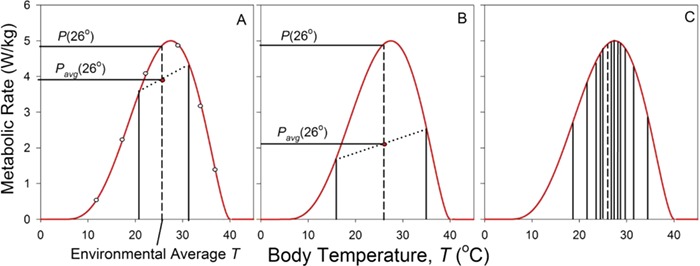
Introducing Jensen’s inequality. (A) The nominal thermal performance function (red line) is fit to empirical measurements of performance at a series of constant temperatures (open dots). If temperature alternates between 21°C and 31°C, average performance (the red dot) is less than performance at the average temperature (26°C, shown by the dashed line). (B) Greater variation in temperature (16°C to 36°C) results in lower average performance. (C) Average performance in a continuously varying thermal environment can be estimated by averaging the performance at *n* temperatures grouped around the average temperature.

Typically, a species’ thermal performance curve is measured through a series of experiments (Fig. 2A). A group of the specified plant or animal is placed at a constant temperature *T* and the average per capita performance (in this example, metabolic rate) is quantified. A second group is then held at a different temperature, its performance measured and so forth (the open dots in Fig. 2A). After a sufficient number of points have been obtained, a function, *P*(*T*), is fitted to the data, and this continuous function is the organism’s nominal thermal performance function. A variety of functions have been used (Angilletta, 2009; Knies and Kingsolver, 2010); here, I use a modified beta function (Dowd et al., 2015). With this function in hand, one can then specify the organism’s performance at any temperature. Ecologists often use performance at the environment’s average temperature as a measure of an organism’s performance in that environment. For instance, in the hypothetical example of Fig. 2A, if an animal’s body temperature is constant at the environmental average of 26°C, its metabolic rate is 4.8 W/kg.

### Jensen’s inequality or the fallacy of the average

There is a basic problem with this approach, however. As I have noted, for most organisms temperature—and thus performance—fluctuates through time, and this fluctuation affects our assessment of the performance we expect of the organism. To see how this works, consider again the thermal performance curve shown in Fig. 2A. Let us assume (unrealistically, but heuristically) that the organism’s body temperature alternates between 21°C at night and 31°C during the day, with 12 h spent in each state. The organism’s average body temperature is thus 26°C (the same as the environment’s average temperature). Performance is low at night and higher during the day, but the organism’s average performance can be calculated graphically by drawing a line between the nighttime and daytime performance values and noting the performance at its midpoint. (If unequal times were spent at nighttime and daytime temperatures, the resulting average would be calculated by sliding the measurement point proportionally along the line connecting the two temperatures.) Because the thermal performance function is convex downward, the average of nighttime and daytime performances, *P_avg_*(26°), is lower than the performance at the average temperature *P*(26°). The fact that average performance when temperature varies differs from performance at average temperature is an example of a general conclusion regarding averages of nonlinear functions known informally as the fallacy of the average and technically as Jensen’s inequality (named for Johan Jensen, a Danish mathematician who explained the phenomenon in Jensen, 1906; Ruel and Ayres, 1999; Denny, 2017). In mathematical terms, if function *g*(*x*) is nonlinear, the average of the function, $\overline{g(x)}$, is not equal to the function of the average, $g\Big(\overline{x}\Big)$:

(1) \begin{equation*} \overline{g(x)}\ne g\left(\overline{x}\right). \end{equation*}

This relationship holds for nonlinear functions of any variable, and Jensen’s inequality is responsible for a wide variety of natural phenomena. For example, in the context of variable atomic dipole strength Jensen’s inequality accounts for the van der Waals forces that stabilize enzymes (De Podesta, 2002). In the context of variable gamete concentration, it explains the ability of sea urchins to reproduce effectively by shedding their gametes into turbulent flow (Crimaldi et al., 2008; Denny, 2017). In the context of performance *P* in the presence of variation in temperature *T* (the focus of this review):

(2) \begin{equation*} \overline{P(T)}\ne P\big(\overline{T}\big). \end{equation*}

In the example of Fig 2A, $\overline{P(T)}$ is less than $P\big(\overline{T}\big)$ because (in the vicinity of 26°C) the performance function is convex downward. If we were to pick two points bracketing an average temperature near *CT_min_*, we would find that the average of the function is larger than the function of the average because there the curve is convex upward.

We now extend this basic analysis. We have noted that alternation in temperature by $\pm$5°C around an average temperature of 26°C results in a decrease in average performance (Fig. 2A). If we increase the variation in temperature to $\pm$10°C, average performance is even lower (Fig. 2B). Generalizing from this theme, the greater the variation in temperature, the greater the effect on average performance. This conclusion can be quantified mathematically. To a first approximation (Chesson et al., 2005; Denny, 2016):

(3) \begin{equation*} \overline{P(T)}\cong P\big(\overline{T}\big)+\frac{1}{2}P^{\prime\prime }{\sigma}^2. \end{equation*}

Here, $P^{\prime\prime }$ is the second derivative of *P* (the nominal thermal performance function) with respect to temperature *T*, taken at the average temperature $\overline{T}$,

(4) \begin{equation*} {P}^{\prime \prime }=\frac{d^2P\big(\overline{T}\big)}{d{T}^2}, \end{equation*}

and ${\sigma}^2$ is the temporal variance of body temperature experienced by the organism. Although it is strictly true only for values of *T* where the first derivative of *P* = 0, one can think of the second derivative as the curvature of the performance function. When curvature is negative, the function is concave downward, and accordingly, Equation 3 tells us that $\overline{P(T)}<P\big(\overline{T}\big)$. If $P^{\prime\prime }$ is positive, $\overline{P(T)}>P\big(\overline{T}\big)$.

The approximation represented by Equation 3 is useful in that it provides a mathematical method for intuiting the effect of variation on average performance. However, the precision of this approximation depends on the range over which temperatures vary about the mean temperature. If this range is small relative to thermal breadth, Equation 3 is reasonably accurate. If the range is large relative to thermal breadth, Equation 3 can be substantially in error. Fortunately, there is an alternative method for calculating $\overline{P(T)}$ that builds on the simple approach of Fig 2A and B (Fig. 2C; Benedetti-Cecchi, 2005). Recall that in Fig 2A and B, we calculated mean performance by averaging nighttime and daytime temperatures. However, we are not limited to picking just two temperatures. Instead, we can choose at random a large number of values from the temperature distribution experienced by a real organism. This distribution can have a wide variety of shapes: skewed to high temperatures, skewed to low temperatures, even bimodal. For simplicity, however, I model thermal variation using a Gaussian distribution with its mean set to that of the organism ($\overline{T}$) and a standard deviation of ${\sigma}_t$ (the subscript *t* denotes variation through time). Values of *T* (*T_i_*, where *i* = 1 to *n*) are picked at random from this distribution and are therefore clustered around $\overline{T}$ (64% of our choices lie within $\pm 1$standard deviation of $\overline{T}$, an effect shown graphically by the vertical lines in Fig. 2C). Average performance at temperature $\overline{T}$ is then estimated as:

(5) \begin{equation*} \overline{P\big(\overline{T}\big)}=\frac{1}{n}\sum_{i=1}^nP\left({T}_i\right). \end{equation*}

Although somewhat more computationally intensive than Equation 3, this approach has the distinct advantage that as long as *n* is large (>30 or so) the method is accurate regardless of the range of temperatures encountered by the organism or the shape of the temperature distribution.

**Figure 3:**
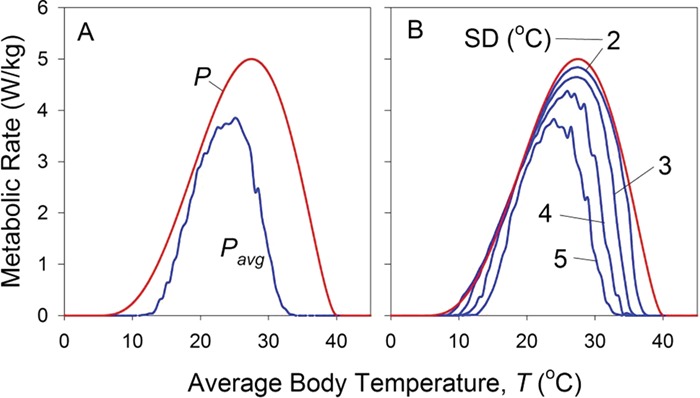
(A) Performance at average body temperature in a variable environment (*P_avg_*, blue line) is lower than nominal performance (red line) and peaks at a lower temperature. (B) The greater the standard deviation of temperature experienced by an individual, the lower its performance and the lower its *T_opt_*.

Equation 5 allows us to calculate average performance at any given average body temperature $\overline{T}$. By repeatedly applying this equation to temperatures across an organism’s entire thermal range, we can specify a new thermal performance function, *P_avg_*($\overline{T}$), that quantifies performance, not under the constant conditions used to specify the nominal performance curve *P*(*T*), but rather performance in the presence of thermal variation (Fig. 3A).

Several conclusions can be drawn from our calculation of *P_avg_*. Performance is lower in the presence of thermal variation than it is under constant conditions, a consequence of Jensen’s inequality. Furthermore, the temperature at which performance peaks in *P_avg_* is less than that for the nominal curve *P*. Lastly, the breadth of the performance curve is reduced. (This is a consequence of our assumption that animals die if at any time their body temperature is less than *CT_min_* or greater than *CT_max_*.) In short, given the shape of the typical nominal performance curve, plants or animals perform less well in the presence of thermal variation than when temperature is held constant.

The magnitude of these effects depends on the extent of variation in temperature (Fig. 3B). The larger the range of temperatures to which an organism is subjected, the larger the standard deviation of its body temperature, the lower its performance and the lower the temperature at which performance peaks.

At this point, it is necessary to inject a note of caution. The conclusions we have reached by applying Jensen’s inequality to the nominal performance curve assume that physiological plasticity and the temporal pattern of thermal variation play no role in an organism’s performance. However, organisms commonly adjust their physiology (acclimatize) in response to temporal fluctuations in temperature, and these adjustments take time (for a review, see Angilletta, 2009). As a result, performance lags behind any shift in temperature by an interval that can vary from hours to days (e.g. Senius, 1975; Williams and Somero 1996; Hoffmann et al., 2003; Sinclair and Roberts, 2005; Healy and Schulte 2012). The resulting hysteresis can jeopardize our calculation of *P_avg_*. For example, our calculations (Equation 5) assume that if an animal’s body temperature abruptly jumps down from 31°C to 26°C, its performance at 26°C is the same as it would be if its temperature instead had abruptly jumped up from 21°C to 26°C. However, because it takes time for an organism’s physiology to adjust to a shift in temperature, it is likely that an organism moving down to 26°C will, for a period of time, retain some of its adjustments to its previous high temperature, while an organism moving up to 26°C will retain some of its adjustments to its previous low temperature. As a result, the two performances at 26°C will initially be different, violating our assumption. We will return to this and other complicating factor in the Caveats and Challenges section below. For the time being, I ignore these complications and accept the general conclusion that thermal variation reduces performance.

This conclusion has been used to predict the effects of climate change for animals at different latitudes. For example, early efforts noted that average terrestrial temperatures are rising more rapidly in temperate and arctic regions than in the tropics, suggesting that temperate and arctic plants and animals should suffer the greater risk from climate change. However, for tropical terrestrial species the thermal performance function is narrow (and therefore tightly curved), while for temperate and arctic species it is broad (and therefore gently curved). Because increased curvature ($P^{\prime\prime }$) accentuates the effects of Jensen’s inequality (Equation 3), effects of temperature fluctuations are greatest for tropical species and in fact are sufficiently large to place them at greater risk than organisms in temperate and arctic regions (Dillon et al., 2010). Indeed, Paaijmans et al. (2013) and Vasseur et al. (2014) predict that the effects of increased temperature variability associated with climate change will outweigh the effects of increased average temperature. It is worth noting, however, that these efforts to predict the consequences of climate change rely on the simplifying assumption that operative body temperature is equal to air temperature. As we have seen, this need not be the case. For this and many other reasons, the large-scale, long-term consequences of thermal variation in individual organisms continue to be an area of active debate and research (e.g. Martin and Huey, 2008; Kingsolver et al., 2015; Dowd et al., 2015; Buckley and Huey, 2016; Kingsolver and Woods, 2016; Koussoroplis et al. 2017).

### Spatial variability

Rather than delve into the intricacies of the debate regarding the effects of thermal variation on individual performance, I instead turn to the effects of thermal variation on a population of individuals. Just as body temperature can vary through time within an individual, *T* can vary in space among individuals, and the amount of this spatial variation often depends on the scale at which it is measured. This fact applies to all populations, but it is convenient to envision it for a particular population that has considerable heuristic value.

The intertidal zone of rocky shores is an exceptionally rigorous environment that has long served as a model system for experimental ecology. On many wave-swept rocky shores, mussels of the genus *Mytilus* live in tightly packed beds, forming what at low tide looks like a carpet across the middle of the shore. As the dominant competitor for space, mussels act as ecosystem engineers and thus play an important role in intertidal community ecology. Because they are sessile, mussels cannot move to escape the thermal stresses imposed by solar heating at low tide, and this lack of behavioural thermal regulation substantially simplifies the task of predicting the effects of spatial variation in temperature.

In light of our earlier discussion of temperature variation in air, one can predict that body temperature varies among mussels in a bed when they are emersed at low tide. A large mussel extending above the bed will intercept more sunshine while shading its smaller neighbours, resulting in a higher temperature in the larger individual. Topographic variation in the rock (which tends to be complex) ensures that different areas of bed are presented to the sun at different angles, again causing spatial variation in *T*, and mussels higher on the shore are exposed to terrestrial conditions for longer than their lower bedmates as the tides ebb and flow, allowing them to both heat and cool for longer periods.

The variation in *T* resulting from these factors depends on the spatial scale at which it is measured. Mussels adjacent to each other are likely to have temperatures that are more similar than mussels a few meters apart, which, because the bed conforms to the complex topography, might have a different orientation relative to the sun. Temperatures are likely to vary even more between mussels separated by tens of meters on different sides of a promontory (Denny et al., 2004).

### 1/*f* noise

The scale-dependence of this sort of spatial variation has received considerable attention from ecologists, and a general picture has emerged (e.g. Halley, 1996; Inchausti and Halley, 2002; Vasseur and Yodsis, 2004; Denny, 2016). Traditionally, the pattern in which a variable (e.g. body temperature) varies through space is described by the power spectral density function (the power spectrum), *S*, which quantifies the manner in which the overall variance (${\sigma}_s^2$ in this case) is distributed among spatial frequencies, *f_s_* (the subscript *s* denotes variation through space). (Spatial frequency is an analogue of temporal frequency. For example, the pressure in a sound wave of a given frequency *f* repeats itself every *t* seconds such that *f =* 1/*t*. Analogously, spatial frequency is the inverse of the distance $\ell$ over which a spatial pattern repeats itself: *f_s_* = 1/$\ell$.) When *S* (which has units of variance per frequency) is plotted as a function of *f_s_*, the overall variance is the area under the curve (Diggle, 1990; Denny, 2016):

(6) \begin{equation*} {\sigma}_s^2={\int}_0^{\infty }S\left({f}_s\right)d{f}_s. \end{equation*}

For a given range of frequencies (say from *a* to *b*), the variance associated with that range is

(7) \begin{equation*} {\sigma}_s^2={\int}_a^bS\left({f}_s\right)d{f}_s. \end{equation*}

Often, *S* decreases exponentially with an increase in spatial frequency (Halley, 1996; Denny, 2016):

(8) \begin{equation*} S\left({f}_s\right)=\frac{k}{f_s^{\beta }} \quad (\beta \geq 0). \end{equation*}

**Figure 4:**
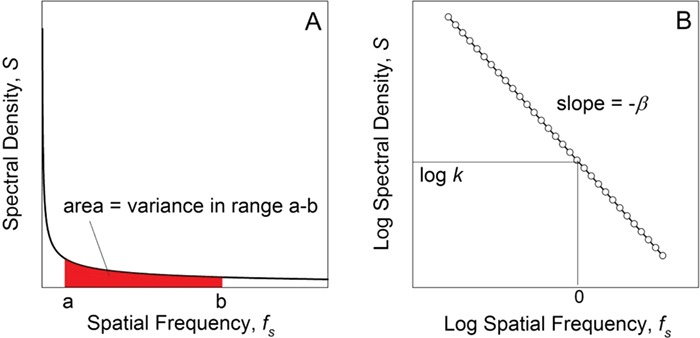
The 1/*f* spectrum. (A) Spectral density *S* decreases exponentially with an increase in spatial frequency *f_s_*. The area under the curve between frequencies a and b is the variance in temperature in this frequency range. (B) The curve from (A) re-plotted on log-log axes with the individual data points shown (open circles). The slope of the line is equal to –*β*, and the value of spectral density at log *f_s_* = 0 is log *k*.

An example of this iconic spectrum is shown in Fig. 4A. $\beta$ controls how variance scales with spatial frequency, and *k* is a coefficient that determines the overall magnitude of variance. $\beta$ and *k* can be estimated from empirical measurements of the variable in question using standard methods of spectral analysis (e.g. Diggle, 1990). In our heuristic example, one would measure the body temperature of mussels at equally spaced intervals along a transect across the bed. If the spectrum calculated from these data conforms to Equation 8, values of log(*S*) are linearly related to the log (*f*_s_) (Fig. 4B), and $\beta$ and *k* can be estimated from the slope and intercept of that line, respectively (Clauset et al., 2009).

Because of the form of Equation 8, this pattern of variation is known as 1/*f* noise (‘one over *f* noise’), where ‘noise’ is an informal expression for ‘variance’. Various values for $\beta$ are given names in an analogy to visible light. If $\beta =0$, variance is the same for all spatial frequencies, analogous to white light, which is composed of equal intensities of all frequencies of electromagnetic radiation (i.e. colors). Thus, a pattern in which $\beta =0$ is known as white noise. If $\beta >0$, low spatial frequencies have more variance than high frequencies, analogous to light that is skewed to the red end of the spectrum, so this common spatial pattern is known as red noise.

Many naturally occurring patterns exhibit reddened 1/*f* noise. For instance, in an exhaustive literature survey, Inchausti and Halley (2002) found that 97% of documented time series of species abundance were characterized by red noise. The elevations of points on a fractal surface are red noise (Hastings and Sugihara, 1993), as are the nucleotide sequences in DNA (Halley, 1996). Most pertinent to the present discussion, on at least one representative shore the spatial pattern of variation in mussel body temperature can be described as reddened 1/*f* noise (Denny et al., 2004).

The utility of 1/*f* noise is most easily grasped if we switch our focus from spatial frequency (the traditional factor used to describe 1/*f* noise) to spatial scale (a more intuitive metric). Recall that *f_s_ =* 1*/*$\ell$, where $\ell$ is the spatial scale at which a pattern repeats itself. Using this relationship, one can manipulate Equation 8 to quantify the overall variance in temperature associated with a given range of $\ell$ (Denny, 2016). For $\beta \ne 1:$

$$ {\sigma}_s^2=\frac{k}{1-\beta}\left({\ell}_{min}^{\beta -1}-{\ell}_{max}^{\beta -1}\right) $$

(9) \begin{equation*} {\sigma}_s=\sqrt{\frac{k}{1-\beta}\left({\ell}_{min}^{\beta -1}-{\ell}_{max}^{\beta -1}\right)} \end{equation*}

For $\beta =1$:

$$ {\sigma}_s^2= kln\left(\frac{\ell_{max}}{\ell_{min}}\right) $$

(10) \begin{equation*} {\sigma}_s=\sqrt{kln\left(\frac{\ell_{max}}{\ell_{min}}\right)} \end{equation*}

**Figure 5:**
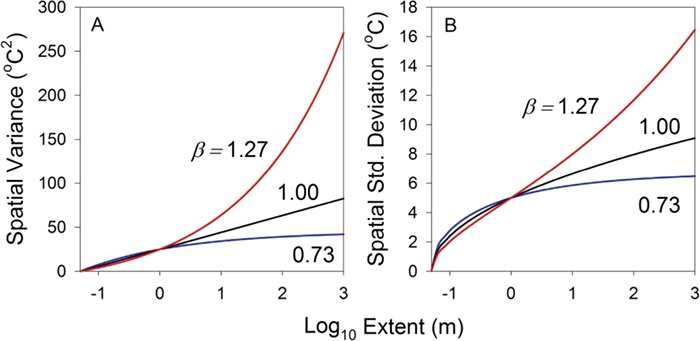
Spatial variation for a 1/*f*-noise process: the longer the extent over which thermal variation is measured (the extent of the measurement), the great the variance (A) and standard deviation (B). In these examples, I have set *k* for each $\beta$ (Equation 8) to the value that yields a variance of 25 (W/kg)^2^ at $\ell$*_max_* = 1 m. Note that for $\beta <1$ variance asymptotes, whereas for $\beta \geq 1$ variance increases without limit.

In these equations, $\ell$*_min_* (the grain of the population) is the smallest scale at which we can measure temperature variation, and $\ell$*_max_* (the extent) is the largest scale of interest. Figure 5 illustrates the manner in which the magnitude of $\beta$ affects thermal variance. For 0 < $\beta <1$, variance increases with increasing range of spatial scale, but asymptotes to a finite value at large scale. For $\beta$ = 1, variance increases linearly without limit, and for $\beta$ > 1, variance likewise increases without limit, but at an accelerating pace. In Fig. 5B, these relationships are expressed as the standard deviation of temperature associated with that extent (${\sigma}_s$) rather than the variance $\big({\sigma}_s^2\big)$.

### Spatially variable performance

We are now poised to incorporate spatial variation of body temperature among individuals into an estimate of population-level performance. The process is conveniently illustrated using our mussel-bed example. We desire to estimate the average metabolic rate of a population of mussels on a particular shore. We begin by measuring *T* for individuals at equally spaced intervals along a short transect (20 m, say) and from the spatial pattern of these individual temperatures we estimate *k* and $\beta$. For the moment, let us assume that the size of an individual mussel (0.05 m) is the smallest spatial scale at which we can measure variation in body temperature; this sets $\ell$*_min_*. Let us further suppose that the population extends for 1 km along the shore, setting $\ell$*_max_*, the extent. With *k*, $\beta$, $\ell$*_min_* and $\ell$*_max_* in hand, we can then use Equations 9 or 10 to calculate ${\sigma}_s$, the standard deviation of temperature among individuals along the shore.

We now return to *P_avg_*, our prediction of average individual performance as a function of thermal variance (Equation 5, Fig. 3A). Recall that this curve quantifies the effect on an individual of temporal variation in temperature. Our task now is to use this curve of average individual performance to estimate the effect of spatial variation in temperature among individuals. We do so by again applying the concept of Jensen’s inequality.

The procedure is the same as before. For a given average temperature among individuals in a population, ${\overline{T}}_{pop}$, we randomly choose *n* temperatures (*T_i_*, where *i* = 1 to *n*) from a Gaussian distribution with a mean of ${\overline{T}}_{pop}$ and the standard deviation ${\sigma}_s$ specified by Equations 9 or 10. For each of these sample temperatures we note the value of *P_avg_*, and then average these values:

(11) \begin{equation*} \overline{P_{avg}\big({\overline{T}}_{pop}\big)}=\frac{1}{n}\sum_{i=1}^n{P}_{avg}\left({T}_i\right). \end{equation*}

**Figure 6:**
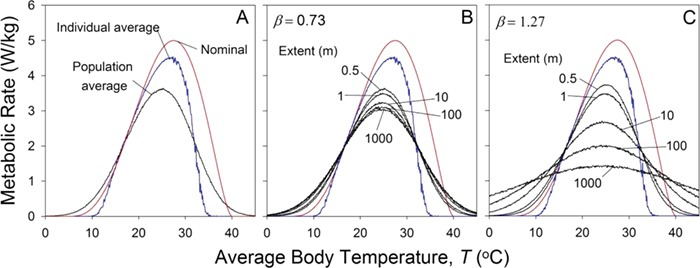
The effect of spatial variance on population average metabolic rate. (A) Spatial variation reduces population average performance below that of individual average performance and increases thermal breadth. (B) For $\beta =0.73$, the effects of increasing the extent of measurement are minimal for extents longer than 100 m. (C) For $\beta =1.27$, the effects of increasing the extent of measurement continue to well beyond 100 m.

The procedure can then be repeated for different values of ${\overline{T}}_{pop}$. The result is shown in Fig. 6A. As expected from Jensen’s inequality and the variation in temperature among individuals, average performance of the population is lower than that of individuals, and the temperature of peak population performance is lower than that of individual performance. However, it may surprise you to see that the thermal breadth of the population is substantially larger than that of the individual. The explanation for this phenomenon is relatively straightforward. Consider a population-scale average temperature at the individual-scale *CT_max_*. If all individuals in the population experienced this temperature, the population’s performance would be zero. However, temperature varies among individuals in the population. Those that experience temperatures > *CT_max_* die (their performance is 0), but those that experience temperatures < *CT_max_* live (with a performance > 0). Thus, even at an average temperature equal to the *CT_max_* of *P_avg_*, the surviving population has an average performance greater than zero. An analogous argument applies to population average temperatures near *CT_min_*. These effects are described in detail by Denny et al. (2011).

The variance-induced decrease in population average peak performance and increase in population average thermal breadth depends on the scale (= extent, $\ell$*_max_*) at which the population is measured. Because we have assumed that thermal variation in mussel beds is 1/*f* noise, the larger the scale at which we view the population, the larger the overall thermal variance and the more profound the decreases in peak performance and increase in breadth.

The effect of spatial scale on population performance is in turn sensitive to $\beta$, the exponent in the 1/*f*-noise equation (Equation 8). $\beta$ has been measured for thermal variation in both marine and terrestrial systems. Steele (1985) suggested that because the high heat capacity and thermal conductivity of water discourage small-scale (i.e. high spatial frequency) variation in temperature in marine systems, they should appear more ‘red’ (a higher $\beta$) than terrestrial systems. This suggestion is borne out in a survey of available data by Vasseur and Yodsis (2004), who found that the $\beta$s of terrestrial systems varied from 0.1 to 0.75 while those of marine systems varied from 0.75 to 1.5. Much work remains, however, before we have a definitive picture of the patterns of spatial variability in temperature. In Fig. 6B, I have used $\beta =0.73$, the value measured for a particular mussel bed by Denny et al. (2004). Because this value is less than 1, ${\sigma}_s$ changes little at large ${\ell}_{max}$, and the performance becomes essentially scale independent for $\ell$*_max_* > 100 m. In Fig. 6B, I set $\beta$ to 1.27 (a value as much above 1 as 0.73 is below). In this hypothetical (but realistic) case, increasing scale continues to have an effect on performance even at large extents.

### Caveats and challenges

The two-step application of Jensen’s inequality outlined here, coupled with the suggestion that spatial variation in individual body temperature scales as 1/*f* noise, provides a heuristic recipe for scaling up from small-scale measurements of temperature to large-scale estimates of population performance, estimates that can be of value to conservation biologists. However, there are several assumptions involved in this method that require closer inspection.

First, when I accounted for the thermal variation encountered by an individual, I assumed that that variation could be accurately modelled by a Gaussian distribution and that the standard deviation of that distribution was the same for all average body temperatures. Neither assumption is likely to be accurate in the real world. Given sufficient empirical data, it would be best to use the distributions of measured body temperature rather than an assumed Gaussian model. At present, such measurements are in short supply, however, so it is uncertain how the use of more realistic temperature distributions will affect average population performance.

An analogous caveat applies to my assumption that inter-individual body temperatures scale as 1/*f* noise. Again, empirical data could come to the rescue. Given sufficient measurements of temperature along an extensive transect, the actual spectrum of thermal variance as a function of spatial frequency could be specified, and these empirical values could be used to more accurately calculate population performance. There is a downside to this approach, however. The assumption of 1/*f* noise allows one to extrapolate from small-scale measurements to much larger scale effects. Without this (or some other) model, extrapolation is not possible, and time- and energy-consuming, large-scale measurements will be required.

These limitations and concerns are particularly acute for mobile organisms whose behaviour can have a strong influence on their body temperature. If, for example, desert lizards hide during the day to avoid overheating, and all choose refuges that allow them to maintain the same temperature, there is little reason to assume *a priori* that the variation among individuals would scale as 1/*f* noise.

And then there is the issue of genetic differences among individuals. In calculating the effect of temperature variation among individuals, I have assumed that every individual in the population conforms to the same nominal thermal performance function. However, genetic variation is likely to manifest as intrinsic differences among individuals in their response to temperature fluctuations. For example, Kuo and Sanford (2009) found substantial variation in thermal tolerance in intertidal snails raised for two generations under identical laboratory conditions. Tolerance varied among individuals collected at a single site, and on average among individuals from latitudinally different sites, effects they attribute to genetics differences. Similar effects have been found in fruit flies (Hoffmann et al., 2003). If the distribution of intrinsic differences among individuals can be measured for organisms, this variation can be modelled in a fashion similar to that of spatial variation. Rather than calculating a single curve of *P_avg_* per Equation 5 and Fig. 3A, a different curve would be calculated for each randomly chosen individual based on the experimentally determined distribution of nominal performance curves in the population. Individual curves could then be sampled randomly to calculate average population performance.

Perhaps the largest caveat regarding the method proposed here concerns the complications introduced by physiology. As noted earlier, an organism’s physiology is constantly adjusting to its environment, but these adjustments take time. As a consequence, the ability to perform at a current temperature can depend on the history of temperatures leading up to the present, and this dependence can be complicated. For example, the *CT_max_* of killifish increases over the course of several days in response to an increase in temperature, but reduction in *CT_max_* in response to a decrease in temperature takes much longer (Healy and Schulte, 2012). Similar physiological hysteresis occurs for the temperature-induced shifts in membrane fluidity in goldfish (Cossins et al., 1977). Repeated stresses can either increase thermal tolerance (‘stress hardening’) or decrease it depending on the organism and the precise timing and intensity of stress (reviewed by Angilletta, 2009). Investigating the complicated effects that history can have on physiology is a major challenge (and a major opportunity) for conservation physiologists as they attempt to predict the consequences of climate change.

Lastly, it is important to note that the ideas developed here in the context of metabolic rate can be applied to other metrics of organismal performance: e.g. growth rate, reproductive output, competitive dominance and predatory capability. And temperature, although important, is just one of many factors that can influence performance. As long as one can measure performance as a function of some specified variable, the method proposed here can be used to estimate first the effect of variation on an individual and then the effect of inter-individual variation on a population.
